# YAP-Mediated Mechanotransduction in Skeletal Muscle

**DOI:** 10.3389/fphys.2016.00041

**Published:** 2016-02-16

**Authors:** Martina Fischer, Paul Rikeit, Petra Knaus, Catherine Coirault

**Affiliations:** ^1^Institut National de la Santé et de la Recherche Médicale, Centre National de la Recherche Scientifique, Center for Research in Myology, Sorbonne Universités Université Pierre et Marie Curie University Paris 06Paris, France; ^2^Institute of Chemistry and Biochemistry, Freie Universität BerlinBerlin, Germany; ^3^Berlin-Brandenburg School for Regenerative Therapies, Charité-Universitätsmedizin BerlinBerlin, Germany

**Keywords:** YAP, mechanotransduction, skeletal muscle, hippo pathway, muscle homeostasis

## Abstract

Skeletal muscle is not only translating chemical energy into mechanical work, it is also a highly adaptive and regenerative tissue whose architecture and functionality is determined by its mechanical and physical environment. Processing intra- and extracellular mechanical signaling cues contributes to the regulation of cell growth, survival, migration and differentiation. Yes-associated Protein (YAP), a transcriptional coactivator downstream of the Hippo pathway and its paralog, the transcriptional co-activator with PDZ-binding motif (TAZ), were recently found to play a key role in mechanotransduction in various tissues including skeletal muscle. Furthermore, YAP/TAZ modulate myogenesis and muscle regeneration and abnormal YAP activity has been reported in muscular dystrophy and rhabdomyosarcoma. Here, we summarize the current knowledge of mechanosensing and -signaling in striated muscle. We highlight the role of YAP signaling and discuss the different routes and hypotheses of its regulation in the context of mechanotransduction.

## Introduction

Mechanotransduction refers to the conversion of mechanical inputs to intracellular biochemical and biophysical signals (Wang et al., 2009). Based on the observation that muscle grows in response to exercise and degrades when underutilized, studies on mechanotransduction have been performed already decades ago in skeletal muscle (Goldberg, 1968; Vandenburgh and Kaufman, 1979).

Current studies on mechanotransduction consider different models. The first relies on the initiation of mechanosignaling via stimulation of “mechanosensors.” These are thought to be adhesive, structural or transmembrane proteins, which could react with conformational changes to applied forces, transmitted by the extracellular matrix (ECM) or neighboring cells. These mechanical stimulations are then integrated into signaling pathways induced by soluble factors and consequently regulate transcriptional changes. In an alternative model, the cell itself is considered a compartmentalized mechanical body with given physical properties such as its viscosity, elasticity or stiffness. Here, the cellular mechanics are mainly defined through the actin, tubulin or septin cytoskeleton, intermediate filaments and the nuclear envelope and nuclear skeleton. These intracellular networks are connected to the ECM through adhesion complexes so that the cellular mechanics are in a permanent coordination with the extracellular constraints. According to this model, mechanical changes are not translated into one specialized mechanosensing pathway but into a simultaneous change in various cell processes, which are regulated by cytoskeletal dynamics, including the activation of signaling pathways.

The IGF-1-Akt-mTOR (Insulin-like growth factor I – protein kinase B/Akt - mammalian target of Rapamycin) pathway has emerged to be the main positive regulator of muscle mass (Sandri, 2008; Miyazaki et al., 2011; Schiaffino et al., 2013) and Myostatin-Smad3 has been identified as the main negative regulator of muscle mass (Wackerhage and Ratkevicius, 2008; Rodriguez et al., 2014). Furthermore, Yes-associated Protein (YAP), a transcriptional coactivator downstream of the Hippo pathway, has been shown to be involved in myogenesis, muscle homeostasis and muscle disorders. In parallel, YAP emerged as a key player in mechanotransduction (Dupont et al., 2011; Wackerhage et al., 2014) and several crosstalks between Akt/mTOR or TGFß/SMAD and the Hippo/Yap pathways have been identified which point to a role of YAP in regulating muscle mass through mechanical cues (Jang et al., 2007; Alarcón et al., 2009; Tumaneng et al., 2012; Grannas et al., 2015).

## YAP

The transcriptional co-activator YAP was first described in 1994 as a 65 kDa binding partner of the Yes protein-tyrosine kinase (Sudol et al., 1995). YAP contains a transcription activation domain (TAD) located at the carboxy-terminal half (Yagi et al., 1999), while the amino-terminal half contains one or two WW domains (Sudol, 1996). These WW domains mediate interactions with proteins containing PPxY motifs. A plethora of different proteins bind to the WW domains of YAP including LATS1/2 (Oka et al., 2008), angiomotin (AMOT) (Zhao et al., 2011) and Smad7 (Ferrigno et al., 2002). In its active state, YAP localizes to the nucleus and regulates the activity of several transcription factors including RUNX, SMAD, p73 and ErbB4 and most importantly TEAD family transcription factors, since YAP and TEAD occupy about 80% of the same genomic loci (Zhao et al., 2008). Prominent target genes of YAP include CTGF, Cyclin D1, AREG, Birc5, and myogenic transcription factor Myf5 (Dong et al., 2007; Zhao et al., 2008; Zhang et al., 2009; Watt et al., 2010).

Together with its paralog TAZ (Transcriptional co-activator with PDZ-binding motif), YAP controls a wide range of cellular functions. During embryogenesis in mice YAP is expressed at all stages from blastocyst to perinatal stage. Homozygous disruption of the YAP allele in mice results in embryonic lethality and causes developmental arrest at E8.5. In contrast, TAZ shows a later onset and is not yet expressed at blastocyst stage (Morin-Kensicki et al., 2006). This indicates a unique role of YAP in embryonic development, for which TAZ does not compensate.

Nuclear YAP activity controls the transcription of genes involved in cell cycle control, typically driving proliferation and survival and inhibiting apoptosis (Dong et al., 2007). Thus, by balancing cell proliferation and death, YAP mediates cell contact inhibition *in vitro* (Zhao et al., 2007) and regulates organ size *in vivo* (Camargo et al., 2007; Dong et al., 2007; Xin et al., 2011). As a regulator of cell-cycle control, YAP misregulation can also lead to tumorigenesis. In several tissues YAP is known to function as an oncogene, while its upstream negative regulators and their adaptors have tumor suppressor function. Furthermore, YAP expression in MCF10A cells induces epithelial to mesenchymal transition (EMT) (Overholtzer et al., 2006). In summary, dysregulated YAP activity has been implicated in a wide range of tumor types including intestinal stem cells, hepatocellular, pancreatic, renal, colorectal, breast, and skeletal muscle cancer (Dong et al., 2007; Tremblay et al., 2014; Patel et al., 2015) which has been further reviewed by S. Plouffe (Plouffe et al., 2015).

Furthermore, YAP is involved in cell fate decisions and serves as a “stemness” factor in different progenitor cell pools of the body. Examples include progenitors in the intestinal crypt, neural progenitor cells in the neural tube, epidermal stem cells and satellite cells of skeletal muscle, where YAP activity promotes proliferation and blocks differentiation (Camargo et al., 2007; Cao et al., 2008; Watt et al., 2010; Schlegelmilch et al., 2011).

YAP also influences cell migration, which is also critical for muscle development and regeneration as activated satellite cells need to migrate out of their niche and along the basal lamina of the myofiber. It was shown, that YAP overexpression in MCF10A or HEK293 cells leads to increased migration and YAP knockdown abolishes migration in T47D cells and renal carcinoma cell lines (Haskins et al., 2014; Schütte et al., 2014; Sorrentino et al., 2014; Moroishi et al., 2015).

YAP capacity to balance proliferation, apoptosis and migration also makes it a regulator of regenerative processes in different tissues including intestine and heart muscle tissue as YAP knockdown severely impairs their regenerative capacity (Cai et al., 2010; Xin et al., 2013). In zebrafish, YAP activity in fin regeneration is based on cell density differences along the regenerating tissue, which leads to a graded control of tissue growth (Mateus et al., 2015).

In brief, YAP is a regulator of the cell cycle and cell fate decisions and consequently of development, organ size and tumorigenesis.

### The hippo pathway and YAP regulation

**Figure 1:**
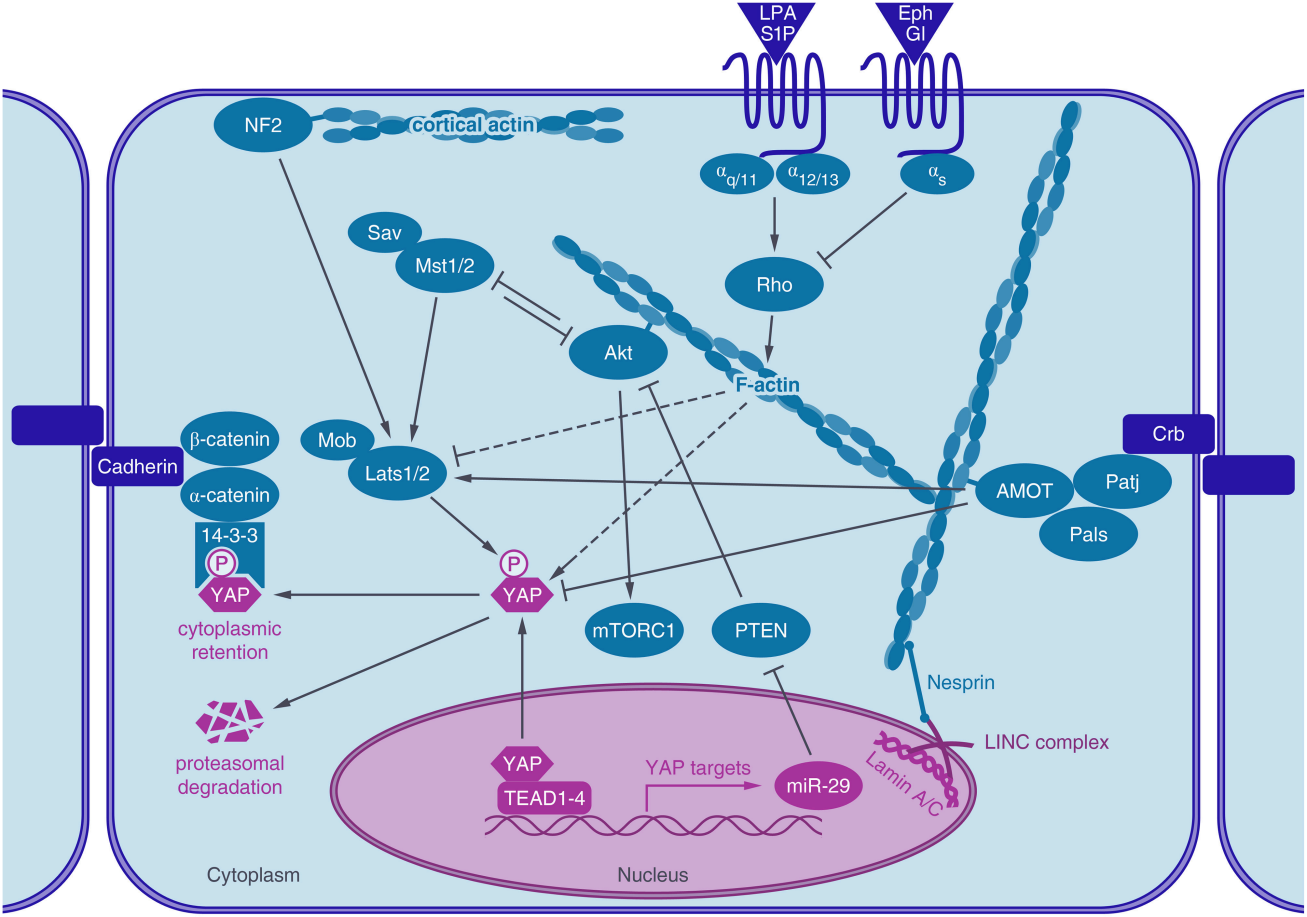
**Actin associated proteins regulate YAP activity**. The transcriptional coactivator YAP shuttles into the nucleus, where it activates TEAD mediated gene expression. After phosphorylation by LATS1/2 kinase, YAP binds to 14-3-3 proteins, leading to its cytoplasmic retention and degradation. YAP activity is regulated by the actin cytoskeleton. Actin stress fibers connect to the lamin meshwork in the nucleus via the LINC-complex. Rho GPTases are regulated by GPCR signaling which in turn regulates actin dynamics and YAP activity (dashed lines). Actin-binding proteins, like angiomotin (AMOT) or neurofibromin 2 (NF2/Merlin) are also known to regulate YAP activity, either through LATS or by direct interaction with YAP. Akt, a key regulator of the IGF-1- mTor pathway also binds to actin stress fibers, crosstalks to the Hippo pathway by interacting with MST1/2 and by YAP induced expression of a microRNA (miR-29) which inhibits the inhibition of Akt by targeting PTEN.

YAP activity is regulated very tightly by the Hippo pathway and a great number of crosstalks, whose interplays are not completely uncovered today (Figure 1). Originally identified by genetic studies in *Drosophila*, the Hippo signaling cascade functions as a highly conserved canonical upstream regulator of YAP activity (Harvey et al., 2003; Wu et al., 2003). At the core of the mammalian pathway is a kinase cassette containing Mammalian Ste20-like 1/2 kinase (MST1/2) and large tumor suppressor 1/2 kinase (LATS1/2) with their adaptors Salvador and MOBKL1A/1B (Mps 1 binder kinase activator-like 1A and 1B) (Figure 1). YAP activity is regulated by phosphorylation, predominantly through five different phosphorylation sites, which are located in HXRXXS consensus motifs for LATS1/2 kinases. The most intensely studied LATS mediated phosphorylation is at serine 127, which leads to binding of 14-3-3 proteins, sequestration of YAP in the cytoplasm and consequently termination of its nuclear activity (Zhao et al., 2007). Phosphorylation at Serine 381 by LATS1/2 however, primes YAP for phosphorylation by casein kinases CK1δ or CK1ε and subsequent ubiquitination via SCF^β*TRCP*^ E3 ubiquitin ligase and proteasomal degradation (Zhao et al., 2010). LATS1/2 kinases are activated by phosphorylation of activated MST1/2 kinases. The activity of MST1/2 and LATS1/2 kinases are further regulated via different trans- and autophophorylation sites. For further reading see the reviews of Visser and Yang (2010) and Rawat and Chernoff (2015).

Moreover, YAP activity is balanced through a negative feedback loop. YAP-TEAD activity induces LATS2 kinase expression and activation of LATS1/2 kinases through Merlin/Neurofibromin 2 (NF2), leading to phosphorylation and inactivation of YAP (Moroishi et al., 2015). Thus, overshooting YAP activity including its tumorigenic potential can be counteracted by this intrinsic regulatory mechanism.

The regulation of YAP by canonical Hippo signaling in mammals was revealed in the context of contact inhibition of proliferation (CIP). Cells grown at low density show nuclear YAP and an inactive Hippo cascade, while at high cell density, the Hippo pathway is switched on and YAP is inactivated via LATS1/2 mediated phosphorylation (Zhao et al., 2007). A specific Hippo receptor as the primary trigger of the Hippo signaling cascade has not been identified yet and the dependence of YAP regulation on Hippo signaling in other contexts has been questioned (Aragona et al., 2013). The main upstream elements regulating YAP activity are discussed in the following paragraphs and are summarized in Figure 1. For further informations see the following publications (Schroeder and Halder, 2012; Johnson and Halder, 2014; Hansen et al., 2015).

### Apicobasal cell polarity, tight junctions, and adherens junctions

YAP/TAZ proteins interact with several components of the Crumbs polarity complex and disrupting the Crumbs complex increases nuclear YAP (Varelas et al., 2010). Furthermore, AMOT proteins, which also localize to the Crumbs complex regulate YAP activity (Paramasivam et al., 2011; Zhao et al., 2011). Also, cadherin-catenin complexes were shown to regulate YAP localization and activity (Schlegelmilch et al., 2011; Silvis et al., 2011). Expression of E-cadherins as well as their association with α- and β-catenin are required for density dependent nuclear exclusion of YAP (Kim et al., 2011). Non-receptor tyrosine phosphatase PTPN14, which plays a role in regulating phosphorylation of β-catenin in adherens junctions (Wadham et al., 2003) was also shown to inhibit YAP activity by promoting its cytoplasmic localization, independently of its phosphatase activity (Michaloglou et al., 2013).

Also Merlin/NF2, another membrane-associated protein, which links cytoskeletal components with proteins in the cell membrane, regulates YAP. The tumor suppressor function of Merlin/NF2, inactivated in Neurofibromatosis type II, acts through the activation of the Hippo cascade most likely by binding and recruiting LATS to the plasma membrane, which in turn promotes LATS phosphorylation by MST (Yin et al., 2013).

### Soluble cues and receptor signaling

YAP can also be regulated through G-protein coupled receptors (GPCRs) and serum starvation inhibits YAP activity via reduced GPCR signaling. G_12∕13_-, G_q∕11_-, and G_i∕o_-coupled receptor agonists (e.g., Lysophosphatidic acid (LPA), sphingosine-1-phosphate (S1P)) activate YAP/TAZ while Epinephrine and Glucagon inhibit YAP/TAZ via G_s_-coupled GPCR signaling (Yu et al., 2012). Recently, the G_12∕13_ subunit was also identified to be activated by the Wnt-FZD/ROR receptor, which would represent an alternative WNT signaling pathway acting through YAP (Park et al., 2015). The regulation of YAP by G-proteins has been shown to be either mediated by the Rho family of GTPases, actin dynamics and LATS (Yu et al., 2012) or PI3-kinase (PI3K) and phosphoinositide-dependent kinase (PDK1) (Gumbiner and Kim, 2014). In addition, epidermal growth factor receptor (EGFR) signaling activates YAP through activation of PI3-kinase (PI3K) and phosphoinositide-dependent kinase (PDK1), which binds to the Hippo core kinases complex (Fan et al., 2013). The neuregulin 1 (NRG1) activated receptor ERBB4, another member of the EGFR family interacts with YAP through its PPxY domain and activates YAP-mediated transcription (Haskins et al., 2014).

## YAP: a key regulator in mechanotransduction

**Figure 2:**
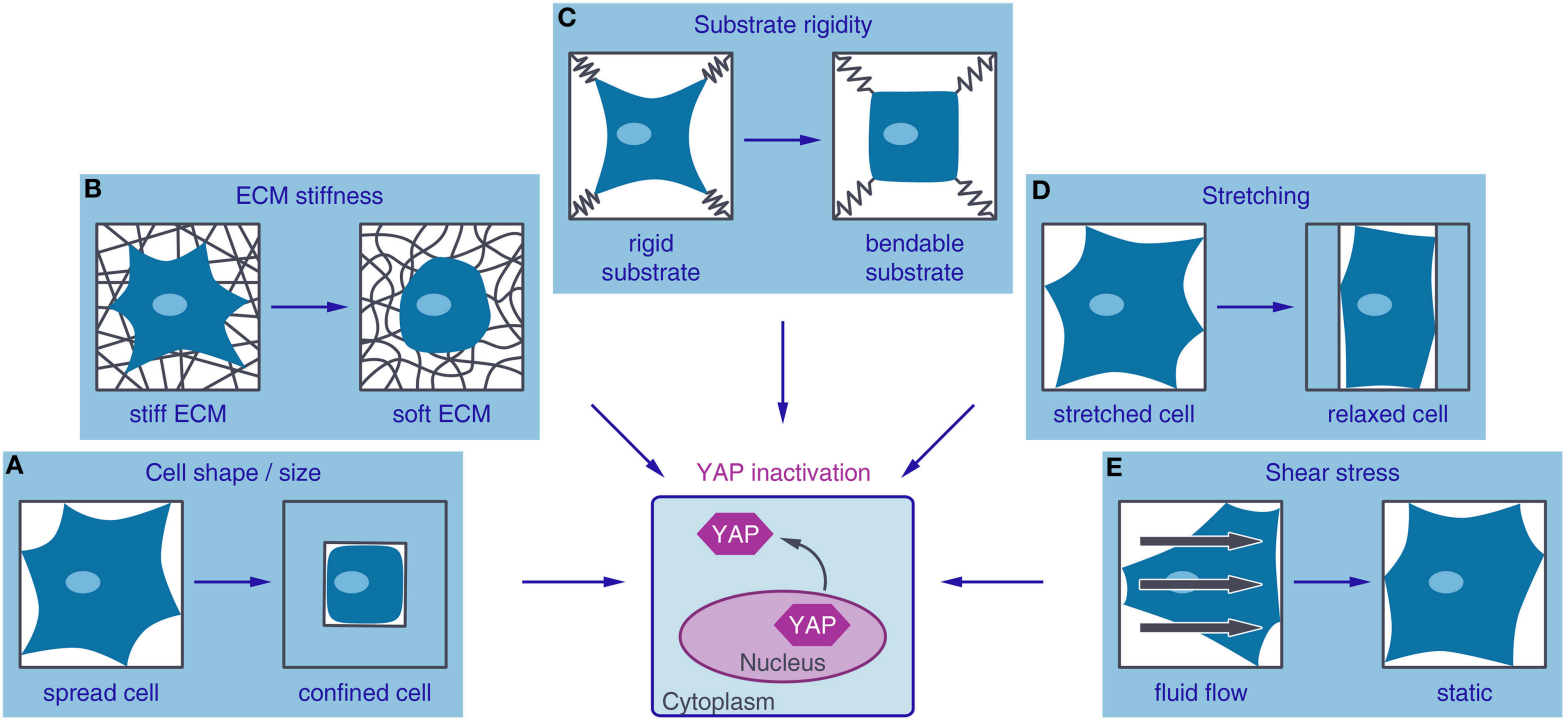
**Different mechanical inputs regulate YAP activity**. YAP (Yes-associated protein) is localized to the nucleus and active under mechanical conditions that lead to high intracellular tension such as a large adhesive area **(A)**, stiff extracellular matrix (ECM) **(B)**, non-bendable substrates **(C)**, cell stretching **(D)**, or fluid shear stress **(E)**. Conditions favoring low contractile forces in the cell, such as small adhesive areas, soft ECM, bendable substrates, relaxation of stretching forces or culture in static media, lead to YAP inactivation by nuclear exclusion.

There is increasing evidence that YAP is a key regulator of mechanotransduction. Pioneering work of Piccolo and co-workers showed that mechanical forces can serve as inputs for the regulation of YAP. By analyzing YAP localization and transcriptional response, they showed YAP activity to be regulated by ECM stiffness, cell-spreading or substrate rigidity (Dupont et al., 2011; Figures 2A–C). Along with these findings, the group of Sasaki proposed a model where cell morphology alone modulates YAP activity. By the use of a microdomain culture system the cell area of a single cell was defined preventing cell-cell contact (Wada et al., 2011). Guan and co-workers further excluded the requirement of focal adhesion sites for the regulation of YAP through cell morphology, by seeding epithelial cells on poly-lysine and attaching them via electrostatic forces (Zhao et al., 2012).

In addition, Piccolo and colleagues showed that YAP can be reactivated in postconfluent culture conditions by stretching the cells, while preventing that these cells lose cell-cell contact (Aragona et al., 2013). The reactivation of YAP by cyclic stretching has also been confirmed on soft surfaces together with an increase in cell spreading, stress fiber formation and proliferation (Cui et al., 2015; Figure 2D). The regulation of YAP by shear stress has only been rarely characterized so far and more research is needed to prove a shear stress-dependent YAP regulation. However, YAP was shown to be activated by fluid shear stress in osteoblasts (Kaneko et al., 2014). Furthermore, increased YAP expression, triggered by fluid shear stress, increased osteogenesis and decreased adipogenesis of hMSCs and initiated dedifferentiation of chondrocytes (Zhong et al., 2013; Figure 2E).

In addition, a mechanical memory was claimed. Long term culture of hMSCs on supraphysiologically stiff substrates can persistently activate YAP and transfer to soft hydrogels cannot inactivate YAP anymore (Yang et al., 2014).

### YAP regulation by intracellular stress

Cell shape and size, ECM stiffness and forces like traction or shear stress all reflect on the cytoskeleton. Published work provides compelling evidence for a critical role of actin dynamics in the regulation of YAP through mechanical cues. Particularly by correlating the activity of YAP with actin stress fiber formation and showing YAP inactivation by the use of F-actin or Rho inhibitors, but not by inhibiting microtubules or Rac1-GEFs (Dupont et al., 2011; Halder et al., 2012; Zhao et al., 2012). Also *in vivo* experiments on *Drosophila* reveal increased actin stress fiber assembly to correlate with YAP nuclear localization and overgrowth of the wing disc (Fernández et al., 2011; Sansores-Garcia et al., 2011). However, the specificity of this effect and the mechanism linking stress fiber formation to YAP activity is controversial and the focus of ongoing research.

Data providing deeper insight into how YAP is regulated by cell morphology and in coordination with cell-contact inhibition in epithelial cells was further published by the Piccolo lab. They found mechanical forces to be the overarching regulators of YAP in a multicellular context. The actin-capping and -severing proteins Cofilin, GapZ, and Gelsolin were identified as gatekeepers, limiting YAP activity in cells which experience low mechanical stress. By depleting actin-capping/severing proteins they showed that increased actin stress fiber formation can restore YAP activity in dense monolayers (Aragona et al., 2013). Assuming the cytoskeleton as the key transducer of mechanical cues into YAP signaling, the role of adaptor proteins like AMOT or other cytoskeletal structures like the tubulin or septin network remain to be further investigated. In regard to skeletal muscle, the role of the structural proteins of the sarcomere needs to be considered as well and their effects on YAP regulation remain to be examined. In the same way, the impact of the polynucleated organization of myofibers in mechanotransduction remain to be investigated.

In addition to changes in cytoskeletal structures, extracellular forces are also transmitted to the nucleus as the cytoskeleton is coupled to the nuclear envelope. The LINC-complex (Linker of Nucleoskeleton and Cytoskeleton), consisting of Nesprin and SUN proteins, thereby connects actin stress fibers to the nucleoskeleton (for further reading, see Lombardi and Lammerding, 2011). Recently, also YAP nuclear translocation was found to be dependent on the force transition to the nucleus through the LINC-complex. By the use of traction force maps, the transfer of the strain to the nucleus was considered essential for YAP localization and activity. Moreover, YAP nuclear relocalization after strain can be prevented by knocking down Nesprin, a protein of the LINC-complex (Driscoll et al., 2015). A-type lamin, an intermediate filament located at the inner nuclear membrane, binds to SUN proteins (Haque et al., 2006) and accumulates at the LINC-complex after applied tension (Guilluy et al., 2014). Consistently, satellite cell-derived myoblasts carrying a mutation in A-type lamin are unable to reactivate YAP after cyclic stretch (Bertrand et al., 2014). Mutations in nucleoskeletal proteins like A-type lamin or Emerin can cause several forms of muscular disorders whose pathophysiology is still not understood.

Transcription factors whose activity is regulated by actin dynamics are already known. For example, megakaryoblastic leukemia 1 (MKL1) binds to G-actin and is released when G-actin polymerizes to form F-actin (Miralles et al., 2003). Moreover MKL-1, together with YAP, has already been implicated in mechanosensing defects of LMNA mutant myoblasts (Bertrand et al., 2014), presumably through modulation of actin dynamics (Ho et al., 2013).

### Role of LATS in mechanotransduction

The dependence of mechanically induced YAP activation on the Hippo core kinases has been challenged. The Guan and Sasaki lab claimed YAP to be regulated by LATS, and LATS to be regulated by stress fibers (Wada et al., 2011; Zhao et al., 2012). By contrast, the Piccolo group found LATS phosphorylation not to be the primary mediator of YAP activity through mechanical cues. YAP and TAZ activity could not be rescued by knockdown of LATS1 and LATS2 after inhibition of actin polymerization (Aragona et al., 2013). Also for the highly discussed adaptor protein AMOT it is not sufficiently clarified if AMOT regulates YAP by either direct binding or via interaction with LATS2 protein (Paramasivam et al., 2011; Zhao et al., 2011). Nevertheless, if actin stress fibers inactivate LATS or sequester a LATS-independent inhibitor of YAP, or both, remains to be clarified.

### YAP crosstalks with other mechanosensitive pathways

YAP interacts with components of other signaling pathways, which play a role in mechanotransduction as well. Canonical TGFβ/BMP signaling acts through SMADs and has been shown to be sensitive to mechanical inputs into the cell (Maeda et al., 2011; Kopf et al., 2014). YAP was shown to bind to activated SMAD1 proteins and to enhance their BMP induced transcriptional activity (Alarcón et al., 2009). YAP also interacts with SMAD2/3 in a TGFβ and cell density dependent manner (Grannas et al., 2015). In addition, YAP nuclear exclusion sequesters SMAD2/3 proteins to the cytoplasm and therefore suppresses TGFβ signaling (Varelas et al., 2010; Narimatsu et al., 2015). Furthermore, YAP interacts with the TGFβ signaling inhibitor SMAD7 (Ferrigno et al., 2002).

Wnt/β-catenin has been implicated in mechanotransduction as well (Huang and Ogawa, 2010; Kang and Robling, 2014). Cytoplasmic YAP inhibits Wnt/β-catenin activity by sequestering β-catenin in the cytoplasm (Imajo et al., 2012) and by interacting with its regulators disheveled and SHP2 (Barry et al., 2013; Tsutsumi et al., 2013). Overexpression of active YAP in mouse cardiomyocytes leads to increased β-catenin activity via the IGF-PI3K-AkT-GSK3β axis (Xin et al., 2011). Furthermore, Protein Kinase C zeta can phosphorylate both YAP and β-catenin to inhibit their nuclear activity (Llado et al., 2015). However, the impact of the crosstalk between YAP and TGFβ/BMP or Wnt/β-catenin signaling on mechanotransduction and its relevance in skeletal muscle homeostasis remain to be elucidated.

## Mechanosensing and -signaling in skeletal muscle

Muscle activity is known to be a major regulator of skeletal muscle mass, with an increase in mechanical loading resulting in muscle hypertrophy, and a decrease in mechanical loading resulting in muscle atrophy (Goldberg et al., 1975). This activity-induced muscle growth has been extensively studied (for recent review see Schiaffino et al., 2013; Piccirillo et al., 2014). Numerous signaling molecules have been identified to be involved and robust literature supports the role of the IGF-1-PI3K-Akt-mTOR pathway as a positive regulator and Myostatin-Smad3 as a negative regulator of muscle mass (Sandri, 2008; Wackerhage and Ratkevicius, 2008; Miyazaki and Esser, 2009; Rodriguez et al., 2014). Also YAP was identified to contribute to the regulation of muscle mass, as overexpression of YAP is sufficient to induce skeletal muscle hypertrophy and the amount of YAP protein is increased in skeletal muscle cells after mechanical overload (Goodman et al., 2015).

Mechanosensitive calcium channels and the kinase domain of titin, a structural protein of the sarcomere, have so far been identified as mechanosensors in skeletal muscle (Lange, 2005; Benavides Damm and Egli, 2014; Bogomolovas et al., 2014). They undergo conformational changes in response to mechanical load and thereby initiate signaling pathways, which regulate muscle mass. However, the detailed mechanisms and involved signaling pathways remain controversial.

### IGF-I-PI3K-Akt-mTOR

Upon contraction, Insulin-like growth factor I (IGF-I) is released by the muscles and acts as an autocrine muscle hormone leading to muscle growth. The importance of the IGF-I-PI3K-Akt-mTOR pathway signaling has been largely confirmed and the multi-protein complex mTORC1 emerged to play a fundamental role in the regulation of skeletal muscle mass by regulating protein synthesis and cell size (Sandri, 2008; Frost and Lang, 2012). The activation of the serine/threonine-specific protein kinase Akt appears to be the crucial determinant of the cellular signaling processes and the transition point between atrophy and hypertrophy (Brooks and Myburgh, 2014). Nevertheless, the regulation of Akt/mTOR, especially its dependence on autocrine IGF-1 stimulation, has been an ongoing discussion point (Hornberger and Esser, 2004; Spangenburg, 2009). Clear evidence has been provided that mechanical loading is sufficient for Akt activation (Nader and Esser, 2001; Bolster et al., 2003; Sakamoto et al., 2003) albeit mTOR signaling can also be mechanically activated in the absence of Akt in the mouse model (Miyazaki et al., 2011). Interestingly, it has been shown that Akt binding to the cytoskeleton is dependent on mechanical stretch (Sawada and Sheetz, 2002). However, Hornberger and colleagues report that the inhibition of actin polymerization did not prevent Akt activation after mechanical strain (Hornberger et al., 2005). However, the mechanisms responsible for the mechanical activation of mTORC1 signaling are not yet fully elucidated and have recently been reviewed by Goodman (2014).

### Crosstalk between YAP and the mTOR/Akt signaling

Since mTOR regulates organ size through cell growth by regulating protein synthesis and the Hippo pathway regulates organ size by regulating proliferation it seems apparent that these pathways are coordinately regulated (Csibi and Blenis, 2012).

Akt has been reported to interact with the Hippo pathway via several routes. Akt has been shown to regulate YAP phosphorylation (Basu et al., 2003) but not by phosphorylating YAP directly (Zhao et al., 2007) but presumably through its interaction with MST1/2. In *Drosophila*, PI3K mediated Akt activity was shown to regulate the phosphorylation of yorkie (Yki), the YAP ortholog, most likely via activation of the MST1/2 ortholog hippo (hpo) or even upstream of hpo (Straßburger et al., 2012; Figure 1). In mammals, MST1/2 was already shown to be a binding partner of Akt and to reduce Akt activity (Cinar et al., 2007). Vice versa, MST1/2 was also found to be inhibited by the interaction with Akt (Jang et al., 2007). Finally, data demonstrate that the phosphorylation of MST1/2 can be induced by the mTOR signaling pathway and restrict MST1/2 function to inhibit cell growth in prostate cancer cells (Collak et al., 2012).

Besides protein-protein interactions, transcriptional crosstalks have been identified. Hippo pathway activity negatively regulates Akt transcription in *Drosophilia* (Straßburger et al., 2012; Ye et al., 2012). In human cell lines, the Hippo pathway was shown to regulate mTOR activity via the microRNA-29 (miR-29). YAP activity leads to the expression of miR-29 which inhibits the translation of PTEN, a phosphatase, which in its active state inhibits Akt (Figure 1). Consistently, YAP overexpression or Lats1/2 knockdown increase mTOR activity in skin sections (Tumaneng et al., 2012). However, YAP overexpression induced muscle hypertrophy was recently shown to act through an mTORC1-independent mechanism (Goodman et al., 2015).

## YAP in skeletal muscle physiology and diseases

The role of YAP in cardiac muscle and its regeneration has emerged as a promising field of research (Papizan and Olson, 2014; Wackerhage et al., 2014; Zhou et al., 2015), but knowledge about YAP function in skeletal muscle is still limited. Interestingly, even before the discovery of YAP protein itself, evidence of its importance in muscle was supported by the identification of the muscle promoter elements MCAT, which are regulated by TEAD family transcription factors and are found in promotors of genes coding for contractile proteins (e.g., β-myosin heavy chain, skeletal α-actin) and regulators of myogenic differentiation (Myf5, Mrf4, myogenin; Mar and Ordahl, 1988; Yoshida, 2008; Ribas et al., 2011; Benhaddou et al., 2012). Furthermore, transgenic overexpression of TEAD-1 in mouse muscle leads to a change in myosin heavy chain isoform expression and therefore to a transition from fast to slow oxidative fiber phenotypes (Tsika et al., 2008). This indicates, that YAP activity regulates the transcription of genes important for muscle development, homeostasis and plasticity.

### YAP in skeletal muscle myogenesis and regeneration

**Figure 3:**
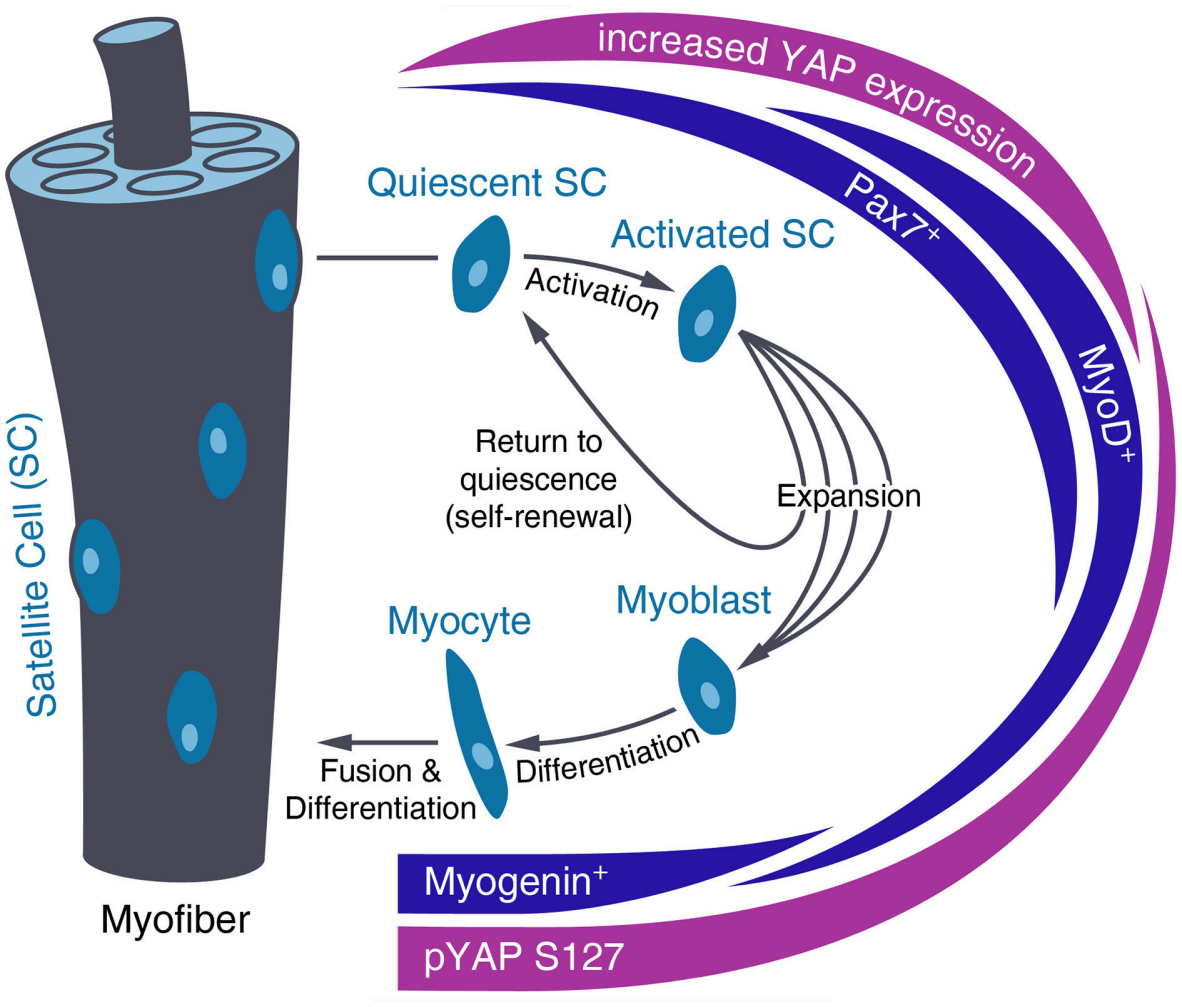
**Regulation of YAP level and activity during satellite cell differentiation**. After activation of quiescent satellite cells (SC), SCs divide and differentiate into myotubes that fuse with existing myofibers or self-renew and return to quiescence. During SC activation, YAP expression increases until this fate decision has been made. In differentiating SCs, YAP is inactivated by increased phosphorylation at Serine 127 (pYAP S127).

For muscle growth and regeneration, activated satellite cells expand, differentiate and then fuse with existing myofibers (Zhang and McLennan, 1994). In their quiescent state, Pax7 expressing muscle stem cells are located between the basal lamina and plasma membrane of the myofiber (Lepper and Fan, 2010). Upon activation, satellite cells start expressing Myf5 and MyoD and proliferate via asymmetric division. Part of this expanded satellite cell pool then undergoes differentiation, marked by myogenin expression and complete downregulation of Pax7. Finally, activated myoblasts fuse with existing myofibers, while the remaining pool of satellite cells self-renews and returns to quiescence (Tedesco et al., 2010; Figure 3).

In this process, high YAP activity promotes proliferation of activated Pax7+ and MyoD+ muscle progenitor cells while YAP inactivation is needed for myogenic differentiation (Figure 3). Changes in YAP activity during satellite cell maturation have been shown *in vitro* and *ex vivo* on murine myoblasts. They show predominantly nuclear YAP during culture and YAP cytoplasmic translocation after myogenic differentiation along with decreased *Yap* mRNA and protein levels and increased YAP phosphorylation (Watt et al., 2010; Judson et al., 2012). Moreover, YAP knockdown reduces proliferation of satellite cell-derived myoblasts but has no impact on progression of their differentiation (Nagata et al., 2006). Further, evidence for the inhibition of skeletal muscle differentiation by YAP activity has been found *in vivo* on *Xenopus laevis* embryos, as YAP overexpression leads to inhibition of MyoD expression (Gee et al., 2011) and on mouse skeletal myofibers, which show reduced YAP levels during postnatal maturation (Watt et al., 2015). *In vitro* overexpression of constitutively active YAP in myoblast precursors results in increased Cyclin D1 and Myf5 expression as well as decreased myogenin, Mef2c and p21 expression, which inhibits terminal myogenic differentiation (Ishibashi et al., 2005; De Falco and De Luca, 2006; Watt et al., 2010).

Regarding YAP regulation, MST1 is activated during myoblast differentiation by caspase3 and active MST1 is needed for proper myoblast differentiation (Fernando et al., 2002). Furthermore, YAP has also been claimed to be involved in the activation of satellite cells by sphingosine-1-phosphate (S1P) mediated YAP activation (Nagata et al., 2006; Yu et al., 2012; Figure 1).

Also, culture conditions show evidence for a YAP dependent regulation of satellite cell differentiation. Established protocols optimized for myogenic differentiation share similarities with those for inactivation of YAP as they recommend high cell density, reduced serum conditions and substrates softer than standard cell culture plastic (Yaffe and Saxel, 1977; Kaushik and Engler, 2014).

### YAP in skeletal muscle homeostasis and disease

In adult skeletal muscle, major Hippo pathway components including YAP are expressed in fast and slow muscle (Watt et al., 2010). In healthy muscle sections, YAP staining is weak and predominantly cytoplasmic, suggesting that YAP does not play a transcriptional role in the function of adult muscle (Crose et al., 2014). However, there are conflicting data on the role of YAP in muscle homeostasis and organ size, including atrophy and hypertrophy. Judson and colleagues report that high levels of a constitutively active YAP mutant drive degeneration, atrophy and necrosis in skeletal muscle fibers by use of a skeletal muscle fiber but not satellite cell specific knock-in mouse model (Judson et al., 2013). Gene expression profiling of these mice show similarities to muscles from mdx mice, a model for Duchenne muscular dystrophy (Hoffman et al., 1987). Interestingly, this muscle wasting phenotype is largely reversible as inactivation of the transgene rescues the degenerative phenotype.

Watt and colleagues on the other hand found YAP as a positive regulator of skeletal muscle size through a TEAD-dependent but mTOR-independent regulation of protein synthesis after knockdown or overexpression of YAP. Furthermore, they report YAP to limit neurogenic atrophy following muscle denervation, since YAP knock out prior to denervation dramatically increased atrophy of muscles (Watt et al., 2015). Goodman and colleagues support the hypertrophic role of YAP in muscle. The chronic mechanical overload model in mouse, which leads to a progressive increase in muscle mass, shows increased YAP expression and phosphorylation. Vice versa, overexpression of YAP in the mouse tibialis anterior leads to hypertrophy (Goodman et al., 2015). Furthermore, increasing muscle mass by blocking myostatin and activin signaling in mice *in vivo* leads to increased total YAP and YAP phosphorylation. Finally, physical exercises also increase YAP phosphorylation levels in mouse limb muscles (Hulmi et al., 2013).

A possible explanations for the contrasting results on the role of YAP in muscle might be the different time points, which have been analyzed, or the use of different YAP mutants, as the constitutive active YAP S127A mutant, in contrast to wild type YAP, cannot be subject to negative feedback regulation.

The upstream regulation of YAP during muscle homeostasis remains poorly characterized so far. By examining neurogenic atrophy, MST1 expression was found to be upregulated in fast- but not slow-dominant muscle and knockout of MST1 attenuated fast-dominant skeletal muscle wasting. Whether YAP phosphorylation and activity are affected here, has not been determined (Wei et al., 2013).

YAP signaling is also implicated in skeletal muscle diseases. Rhabdomyosarcomas are cancers of skeletal muscle tissue that are divided into different subtypes, the two main ones being embryonal rhabdomyosarcoma (eRMS) and alveolar rhabdomyosarcoma (aRMS). Levels of YAP phosphorylation show high variability between different RMS cell lines. Total YAP protein levels, however, are elevated in RMS cells and histological RMS tumor sections show increased nuclear YAP stainings (Crose et al., 2014). Overexpression of constitutively active YAP in activated but not quiescent satellite cells leads to muscle tumors similar to eRMS. *In vitro* and *in vivo* YAP knockdown experiments revealed that lowering YAP expression in human eRMS can rescue tumorigenicity (Tremblay et al., 2014). aRMS is characterized by expression of the paired box 3-forkhead box protein O1 (PAX3-FOXO1) (Galili et al., 1993; Shapiro et al., 1993). PAX3-FOXO1 directly upregulates RASSF4 in aRMS cells and tumors, which associates with MST1 and inhibits its tumor suppressor function, leading to tumorgenesis (Crose et al., 2014).

Furthermore, muscles of mdx mice show elevated levels of phosphorylated and total YAP protein (Judson et al., 2013). Finally, the first evidence of an involvement of YAP-mediated mechanosensing defects in patients with LMNA-related congenital muscular dystrophy has been reported recently (Bertrand et al., 2014). Along with several defects in the organization of the cytoskeleton, YAP was found not to respond to the changing mechanical properties of their environment. While YAP is excluded from the nucleus in soft environment in healthy control cells, patient derived cells maintain nuclear YAP localization in soft environment.

## Concluding remarks and perspectives

YAP has emerged as an important player in mechanotransduction, transmitting mechanical cues into a transcriptional cell response. At the same time, YAP has been shown to be involved in skeletal muscle development and regeneration, as YAP contributes to the regulation of activation, proliferation and differentiation of satellite cells. Beyond that, YAP signaling is also important in adult skeletal muscle homeostasis as misregulation can lead to atrophy or hypertrophy and aberrant YAP activities have been observed in disease states, including skeletal muscle dystrophies. Adult muscle homeostasis again is mainly regulated by muscle activity, which is a mechanical cue itself. Akt-mTOR signaling is widely accepted as the main regulatory pathway defining muscle mass, but the autocrine activation of this pathway by IGF-1 is controversial and several crosstalks to YAP have been identified. Precise mechanisms by which YAP is regulated by mechanical cues are still unknown. Cytoskeletal and presumably also nucleoskeletal tension, in particular actin dynamics and Rho signaling, have been identified as important players of mechanotransduction on YAP, but the detailed mechanism still remains to be elucidated. Uncovering this mechanism, also in regard to the specially organized cytoskeleton of postmitotic myofibers, could reveal important insights into our understanding of muscle homeostasis and subsequently into the physiopathology of muscle diseases.

## Author contributions

MF conceived and wrote the article, PR contributed to the writing and designed the figures. PK and CC revised the article. All authors approved the work for publication.

### Conflict of interest statement

The authors declare that the research was conducted in the absence of any commercial or financial relationships that could be construed as a potential conflict of interest.
